# Hybrid Beamforming for Reduction of Inter-Beam Interference in Millimeter-Wave Cellular Systems

**DOI:** 10.3390/s18020528

**Published:** 2018-02-09

**Authors:** Sung Joon Maeng, Su Ho Park, Yong Soo Cho

**Affiliations:** School of Electrical and Electronics Engineering, Chung-Ang University, Seoul 06974, Korea; aod0527@naver.com (S.J.M); psooh1219@naver.com (S.H.P.)

**Keywords:** millimeter-wave, cellular system, hybrid beamforming, multicell multiuser, inter-beam interference

## Abstract

In millimeter-wave (mm-wave) cellular systems, beamforming antennas are necessary at both the base station (*BS*) and mobile station (*MS*) to compensate for high attenuation in mm-wave frequency bands and to extend the transmission range. The beamforming antennas also allow each *BS* to serve a number of *MS*s simultaneously, providing a substantial gain in system capacity. In space-division multiple access (SDMA) systems, the challenge is the inter-beam interference (IBI) caused by adjacent beams that are formed by the *BS* in the same cell and *BSs* in neighboring cells. The beams that are formed toward *MSs* in each cell may generate significant interference to *MSs* in neighboring cells, especially for MSs at the cell boundary. In this paper, we propose four different digital precoding techniques (Type-1, Type-2, Type-3, and Type-4) to reduce IBI in mm-wave cellular systems with a hybrid beamformer. Simulation results show that the proposed techniques can reduce the IBI in mm-wave cellular systems effectively, compared with a single-cell multiuser case.

## 1. Introduction

Mobile traffic has been increasing markedly owing to the growth of various devices such as smart phones and sensor nodes. Especially, the sensor nodes such as complementary metal-oxide semiconductor (CMOS) sensors and charge-coupled device (CCD) cameras require a high data rate transmission. By 2020, the mobile traffic volume is expected to increase by more than 1000 times because of the increase in smart phones and sensor networks [1]. In order to support such a considerable increase in the mobile traffic, the 30–300 GHz millimeter-wave (mm-wave) frequency band supporting a wide bandwidth is considered as a possible candidate for 5G mobile communication systems with antenna arrays for directional beamforming at both the base station (*BS*) and the mobile station (*MS*) [2,3]. The array gain obtained with the directional beamforming extends the coverage of the mm-wave systems that suffer from a high path loss owing to a high carrier frequency [4,5,6]. Because of the small wavelength of the mm-waves, antenna arrays can be easily installed in the *MSs*.

Owing to significant costs in the radio frequency (*RF*) chain and the power consumption, analog beamforming is currently preferred over digital beamforming for mm-wave communication systems. In mm-wave communication systems with analog beamforming, switched beamforming techniques with a set of predefined angles are usually used for transmit-receive (Tx-Rx) beamforming. Analog phase shifters are typically used to implement analog beamformers in the *RF* domain. However, the use of analog phase shifters places a constant modulus constraint on the elements of the *RF* beamformer. The limitations of analogue beamforming are the difficulty of controlling signal amplitude and the low-resolution signal phase control [7]. In order to overcome the issues faced by digital and analog beamforming, an alternative beamforming architecture, known as hybrid beamforming, has been introduced [8,9,10,11]. In hybrid beamforming, beamforming is partitioned into analog and digital domains. Because of the additional digital processing, more degrees of freedom are available when designing the hybrid precoder compared to analog-only beamformers. In addition, hybrid beamforming allows the support of multi-stream and multiuser transmissions in a flexible way.

The Tx–Rx beamforming technique using the mm-wave frequency band has already been standardized in IEEE 802.11ad and IEEE 802.15c standards to provide a multigigabit-per-second data rate [12,13,14]. In addition, the pre-5G specification for 5G mm-wave cellular systems, called KT Pyeongchang 5G Technical Specification (5G-SIG), was released in 2016 [15,16]. 5G-SIG, which was approved by leading global wireless communication companies, was prepared to demonstrate 5G pilot services for the Pyeongchang Winter Olympic Games in February 2018. Mm-wave communication is also being considered for enhanced mobile broadband (eMBB) in New Radio (NR) currently being standardized for next generation cellular systems [17]. Both analog beamforming and hybrid beamforming are allowed in the pre-5G specification and NR standard.

In [18], a cell selection technique was considered for mm-wave cellular systems with hybrid beamforming in the initialization stage. In general, the serving cell and best beam pair are selected based on the measurement results obtained by all possible Tx-Rx beam pairs of candidate cells in the initialization stage. A beam pair with maximum signal-to-noise ratio (SNR) is selected as the best beam pair and serving cell. However, the selected cell and beam pair may not be optimal for hybrid beamforming system because the effect of the multipath channel is not considered in the initialization stage. In the paper, a cell selection technique was proposed for mm-wave cellular systems with hybrid beamforming in the initialization stage, considering the hybrid beamforming structure in data transmission stage.

In the data transmission stage, the beamforming antennas allow each *BS* to serve a number of *MSs* simultaneously, providing a substantial gain in system capacity. The challenge pertaining to space-division multiple access (SDMA) systems is the inter-beam interference (IBI) caused by the beams formed by the *BS* for multiuser service. In the case of conventional low-frequency systems, digital precoding techniques are used to reduce the co-channel interference of other users who utilize full channel information in the baseband [19]. However, in mm-wave systems, the implementation of a full digital beamformer with precoder is difficult because of the associated high cost and large power consumption. In addition, it is difficult to obtain full channel information at the *BS* because of the large training and feedback overhead that are required in mm-wave systems with a large number of antennas. In [20,21,22,23], hybrid precoding techniques for multiuser mm-wave systems are proposed to reduce computational complexity in the design of a digital precoder using sparse characteristics of the mm-wave channel as well as to reduce beam training and feedback overhead. However, to date, the design of hybrid precoders has been focused on the reduction of IBI in single-cell multiuser mm-wave systems.

In this paper, we propose precoding techniques for mm-wave systems with a hybrid beamformer in multicell multiuser environments. Unlike single-cell multiuser environments, an *MS* in a multicell environment may receive IBI not only from a serving cell but also from neighboring cells. The beams formed toward *MSs* in the serving cell may generate a large amount of interference in the direction of *MSs* in neighboring cells, especially for *MSs* at the cell boundary. In this paper, we propose four different digital precoding techniques (Type-1, Type-2, Type-3, and Type-4) to reduce IBI in mm-wave cellular systems with hybrid beamforming. Then, we analyze pros and cons of the proposed precoding techniques for reduction of IBI in multicell multiuser environments. We compare the performances of the four proposed precoding techniques in terms of the achievable rate and bit error rate (BER). In addition, for the design of the proposed precoders, we need to estimate the channels between the *MS* and neighboring *BSs* as well as the channel between the *MS* and the serving *BS*. The channel estimation technique which can reduce the processing time in multicell environment is discussed for the design of the proposed precoders.

In Section 2, we propose four different digital precoding techniques for mm-wave systems with a hybrid beamformer in multicell multiuser environments. We derive achievable rates of the four different precoders, and compare pros and cons of the precoders for reduction of IBI in multicell multiuser environments. Also, a channel estimation technique appropriate for the design of the proposed precoders is discussed. Then, in Section 3, we verify the performances of the proposed techniques by performing computer simulations using a simple model of an mm-wave cellular system. Finally, we conclude the paper in Section 4.

## 2. Hybrid Beamforming Techniques for a Multicell Multiuser Environment

**Figure 1:**
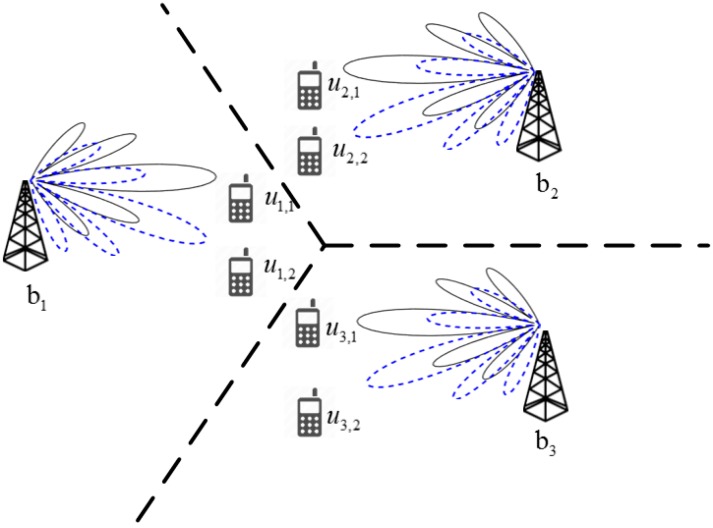
An mm-wave system with a hybrid beamformer in a multicell multiuser environment.

As shown in Figure 1, the signal received at an *MS* in an mm-wave system with a hybrid beamformer under a multicell multiuser environment can be expressed as follows:

(1) $$y_{u_{n,m}}^{}={\left(w_{RF}^{u_{n,m}}\right)}^{*}\{\sum_{l=1}^{B}\left(H_{u_{n,m}}^{b_{l}}F_{RF}^{b_{l}}F_{BB}^{b_{l}}s^{b_{l}}\right)+n_{u_{n,m}}\}$$

where $y_{u_{n,m}}^{}$ is the signal received at the *MS* $u_{n,m}$, which is given by a superposition of signals transmitted from neighboring *BSs* $b_{1},\;b_{2},\;\cdots ,\;b_{B}$. In addition, $u_{n,m}$ is the *m*-th *MS* served by the $b_{n}$-th *BS*, B is the number of neighboring *BSs*, $w_{RF}^{u_{n,m}}$ is the *RF* combiner of *MS* $u_{n,m}$, $H_{u_{n,m}}^{b_{l}}\in {\mathbb{C}}^{N_{MS}\times N_{BS}}$ is the channel between the *BS* $b_{l}$ and *MS* $u_{n,m}$, and $n_{u_{n,m}}$ is the noise component. $F_{RF}^{b_{l}}\in {\mathbb{C}}^{N_{BS}\times L_{BS}},$ $F_{BB}^{b_{l}}\in {\mathbb{C}}^{L_{BS}\times M_{BS}},\;\mathrm{and}\;s^{b_{l}}\in {\mathbb{C}}^{M_{BS}\times 1}$ denote the *RF* precoder, baseband precoder, and symbol transmitted from *BS* $b_{l}$, respectively. Here, $N_{BS},\;L_{BS},\;\mathrm{and}\;M_{BS}$ denote the number of antenna arrays, number of *RF* chains, and number of streams, respectively, all at the *BS*. The analog precoder is called the *RF* precoder, while the digital precoder is called the baseband precoder. Moreover, the transmitted symbol $s^{b_{l}}$ is assumed to be circular [24]. Then, the signal transmitted from the *BS* $b_{n}$ with a hybrid beamformer can be expressed as

(2) $$x^{b_{n}}=F_{RF}^{b_{n}}F_{BB}^{b_{n}}s^{b_{n}}$$

The *RF* precoder $F_{RF}^{b_{n}}$ is usually implemented by a phase shifter. Its entry is given by $\frac{1}{\sqrt{N_{BS}}}e^{j\varphi }$, where φ is the quantized angle of the phase shifter. A ray-based channel model is normally used for mm-wave channels because of a strong line-of-sight (LOS) path component and limited scattering components. Here, we ignore the effect of carrier frequency offset (CFO) and time delays of multi-path channel [25,26]. The delay term for each multipath component is ignored for notational simplicity because, in the following sections, only dominant path (LOS) is considered for the design of proposed precoders. In this paper, it is assumed that each *BS* is equipped with multiple beamforrming antennas and serves a number of *MSs* simultaneously. However, only one beam is formed in the direction of LOS path for each *MS*. Then, the channel with V paths can be expressed as

(3) $$H_{u_{n,m}}^{b_{n}}=\sqrt{\frac{N_{BS}N_{MS}}{V}}\sum_{v=1}^{V}\alpha_{u_{n,m},b_{n},v}a_{MS}\left(\phi_{MS}^{u_{n,m},b_{n},v},\theta_{MS}^{u_{n,m},b_{n},v}\right)a_{BS}{}^{*}\left(\phi_{BS}^{u_{n,m},b_{n},v},\theta_{BS}^{u_{n,m},b_{n},v}\right)$$

where $H_{u_{n,m}}^{b_{n}}$ is the channel matrix between the *BS* $b_{n}$ and *MS* $u_{n,m}$, and $\alpha_{u_{n,m},b_{n},v}$ is the gain of the v-th path between the *BS* $b_{n}$ and *MS* $u_{n,m}$. In addition, $a_{BS}\left(\phi_{BS}^{u_{n,m},b_{n},v},\theta_{BS}^{u_{n,m},b_{n},v}\right)\;\mathrm{and}\;a_{MS}\left(\phi_{MS}^{u_{n,m},b_{n},v},\theta_{MS}^{u_{n,m},b_{n},v}\right)$ denote the antenna array response vectors of the v-th path at the *BS* $b_{n}$ and *MS* $u_{n,m}$, respectively.

When a uniform planar array (UPA) is used, the antenna response vector is given by

(4) $$\begin{array}{l} a_{BS}\left(\phi_{BS}^{u_{n,m},b_{n},v},\theta_{BS}^{u_{n,m},b_{n},v}\right)=\frac{1}{\sqrt{N_{BS}}}{\left[\begin{array}{l} 1,\cdots ,e^{j\frac{2\pi d}{\lambda }(e\cos(\phi_{BS}^{u_{n,m},b_{n},v})\sin(\theta_{BS}^{u_{n,m},b_{n},v})+q\sin(\phi_{BS}^{u_{n,m},b_{n},v})\sin(\theta_{BS}^{u_{n,m},b_{n},v}))},\cdots \\ ,e^{j\frac{2\pi d}{\lambda }((E-1)\cos(\phi_{BS}^{u_{n,m},b_{n},v})\sin(\theta_{BS}^{u_{n,m},b_{n},v})+(Q-1)\sin(\phi_{BS}^{u_{n,m},b_{n},v})\sin(\theta_{BS}^{u_{n,m},b_{n},v}))} \end{array}\right]}^{T}\\ a_{MS}\left(\phi_{MS}^{u_{n,m},b_{n},v},\theta_{MS}^{u_{n,m},b_{n},v}\right)=\frac{1}{\sqrt{N_{MS}}}{\left[\begin{array}{l} 1,\cdots ,e^{j\frac{2\pi d}{\lambda }(e\cos(\phi_{MS}^{u_{n,m},b_{n},v})\sin(\theta_{MS}^{u_{n,m},b_{n},v})+q\sin(\phi_{MS}^{u_{n,m},b_{n},v})\sin(\theta_{MS}^{u_{n,m},b_{n},v}))},\cdots \\ ,e^{j\frac{2\pi d}{\lambda }((E-1)\cos(\phi_{MS}^{u_{n,m},b_{n},v})\sin(\theta_{MS}^{u_{n,m},b_{n},v})+(Q-1)\sin(\phi_{MS}^{u_{n,m},b_{n},v})\sin(\theta_{MS}^{u_{n,m},b_{n},v}))} \end{array}\right]}^{T} \end{array}$$

where $\phi_{BS}^{u_{n,m},b_{n},v}\;\mathrm{and}\;\theta_{BS}^{u_{n,m},b_{n},v}$ denote the azimuth and elevation angles of departure (AoD), and $\phi_{MS}^{u_{n,m},b_{n},v}\;\mathrm{and}\;\theta_{MS}^{u_{n,m},b_{n},v}$ denote azimuth and elevation angles of arrival (AoA), respectively. $N_{MS}$ denotes the number of antenna arrays at the *MS*. In addition, d is the distance between antenna elements, and e (0, ⋯, E−1) and q (0, ⋯, Q−1) are indices of the two axes formed in the antenna array plane.

In a multiuser communication system, multiple antennas allow the *BS* to transmit multiple data streams to a number of *MSs* simultaneously. In the case of conventional low-frequency communication systems, many different precoding techniques such as channel inversion, block diagonalization, dirty paper coding (DPC), and Tomlinson–Harashima precoding (THP) have been used to reduce the co-channel interference in multiuser systems under the assumption that full channel information is available at the *BS* [19]. However, in mm-wave systems, it is difficult to obtain full channel information at the *BS* because mm-wave systems with a large number of antennas require large training and feedback overhead. In [20,21,22,23], a two-stage hybrid precoding algorithm for multiuser mm-wave systems is proposed to reduce the computational complexity using the sparse nature of mm-wave channels. In the first stage, Tx–Rx beamforming is performed in the analog (*RF*) domain to maximize the desired signal power, ignoring the IBI among *MSs*. In the second stage, the digital precoder is designed to minimize IBI among *MSs*. The computational complexity and feedback overhead can be significantly reduced by dividing the calculation of the precoder into two stages. However, in a multicell multiuser environment, the *MS* may receive IBI not only from a serving *BS* but also from neighboring *BSs*, because the beams formed toward *MSs* in the serving cell may generate significant interference to *MSs* in neighboring cells, especially for *MSs* at the cell boundary. In this paper, we propose digital precoding techniques that can reduce IBI in mm-wave cellular systems with a hybrid beamformer, considering interference signals not only from the serving cell but also from neighboring cells. In the proposed technique, we also use the two-stage approach to reduce computational complexity and feedback overhead.

We made the following three assumptions in the design of the proposed digital precoder. First, the first stage of Tx–Rx beamforming has been successfully performed in the analog (*RF*) domain, ignoring the IBI among *MSs*. Thus, in this paper, we focus on the design of a digital precoder (second stage) that can reduce IBI from neighboring cells as well as the serving cell. Second, there is no information loss in the feedback channel in which the effective channel values estimated by *MSs* are transmitted. Third, the effective channel information transmitted by *MSs* is shared with neighboring *BSs* through a backhaul link without any information loss.

**Figure 2:**
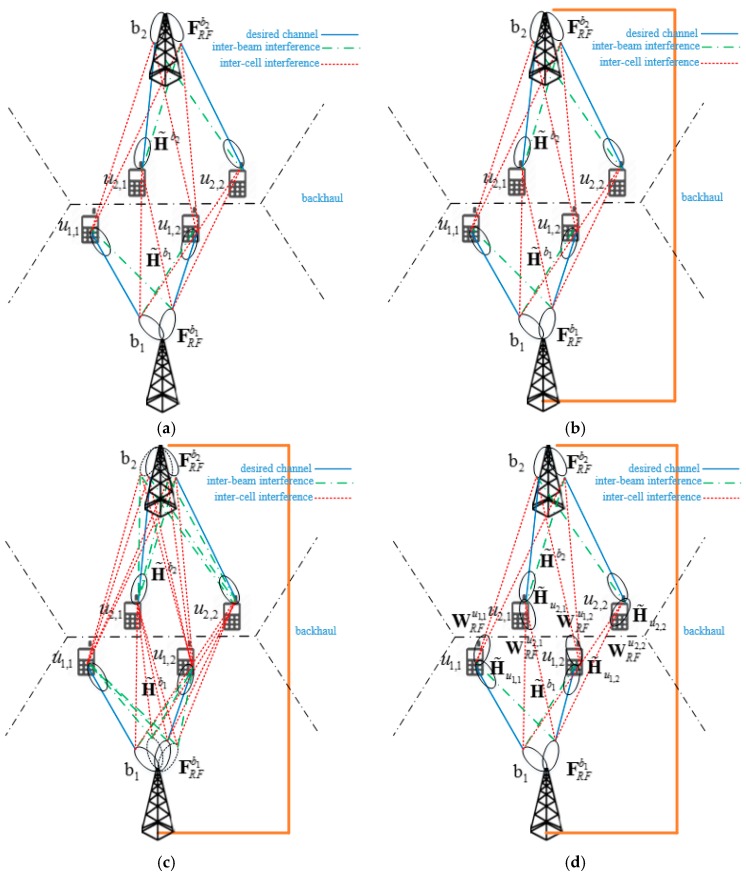
Four different types of digital precoding techniques for reduction of inter-beam interference (IBI) in mm-wave cellular systems with a hybrid beamformer. (**a**) Type-1; (**b**) Type-2; (**c**) Type-3; (**d**) Type-4.

Figure 2 shows four different types of digital precoding techniques for IBI reduction in mm-wave cellular systems with a hybrid beamformer. In this figure, the lines between *BSs* and *MSs* show the directions of interferences as well as the beam directions between the *BS* and desired *MSs*. Here, solid lines represent the beam directions between the *BS* and desired *MSs*. The broken and dotted lines represent inter-beam and inter-cell interferences, respectively, when analog beams are formed in the direction of desired *MS*.

### 2.1. Type-1 Precoder

In a Type-1 precoder, the previous digital precoder developed for a single-cell multiuser environment is directly applied to a multicell multiuser environment. In this case, analog beams are formed in the direction of *MSs* in each cell, as shown in Figure 2a. Beam direction in this figure is obtained after completion of the first stage. Here, the solid line between the left beam of *BS* $b_{1}$ and the *MS* $u_{1,1}$ represents the beam direction between the serving *BS* ($b_{1}$) and desired *MS* ($u_{1,1}$). The solid line between the right beam of *BS* $b_{1}$ and the *MS* $u_{1,2}$ represents the beam direction between the serving *BS* ($b_{1}$) and desired *MS* ($u_{1,2}$). The broken line between the right beam of *BS* $b_{1}$ and the *MS* $u_{1,1}$ represents the direction of inter-beam interference in the serving cell. The inter-beam interference is mainly caused by the sidelobe of analog beam formed toward *MS* $u_{1,2}$ in the serving cell. The dotted lines between two beams of neighboring *BS* $b_{2}$ and the *MS* $u_{1,1}$ represent the directions of inter-cell interference. The channel coefficients associated with these lines correspond to the elements of the effective network channel matrix. In a Type-1 precoder, the *RF* precoder in each cell is given by

(5) $$F_{RF}^{b_{n}}=A_{BS}^{b_{n}}=\left[a_{BS}^{u_{n,1}}\left(\phi_{BS}^{u_{n,1}},\theta_{BS}^{u_{n,1}}\right),\;a_{BS}^{u_{n,2}}(\phi_{BS}^{u_{n,2}},\theta_{BS}^{u_{n,2}}),\cdots ,\;a_{BS}^{u_{n,U}}\left(\phi_{BS}^{u_{n,U}},\theta_{BS}^{u_{n,U}}\right)\right]$$

where $F_{RF}^{b_{n}}$ is the *RF* precoder of the $b_{n}$-th *BS* and $A_{BS}^{b_{n}}$ is the array response matrix of the $b_{n}$-th *BS*. We assumed that the number of *MSs* served by a *BS* is U. That is, a *BS* transmits multiple streams simultaneously to U *MSs* using the *RF* precoder obtained in the first stage. Here, the *MSs* served by the $b_{n}$-th *BS* are denoted by $u_{n,1},u_{n,2},\cdots ,u_{n,U}$. However, in a multicell multiuser environment, a new channel model is needed to take into account the interference terms transmitted from neighboring *BSs*. Here, we define an effective network channel of $b_{n}$, which can be viewed as the effective channel matrix between the *BS* $b_{n}$ and all *MSs* in neighboring cells (including the serving cell). The term “effective” is used to indicate that the *RF* precoder of the *BS* and the *RF* combiner of the *MS* are included in the channel model. From the perspective of the *BS* $b_{n}$, an effective network channel can be expressed as

(6) $${\tilde{H}}^{b_{n}}=\left[\begin{array}{c} {\left(w_{RF}^{u_{1,1}}\right)}^{*}H_{u_{1,1}}^{b_{n}}F_{RF}^{b_{n}}\\ {\left(w_{RF}^{u_{1,2}}\right)}^{*}H_{u_{1,2}}^{b_{n}}F_{RF}^{b_{n}}\\ \vdots \\ {\left(w_{RF}^{u_{1,U}}\right)}^{*}H_{u_{1,U}}^{b_{n}}F_{RF}^{b_{n}}\\ {\left(w_{RF}^{u_{2,1}}\right)}^{*}H_{u_{2,1}}^{b_{n}}F_{RF}^{b_{n}}\\ \vdots \\ {\left(w_{RF}^{u_{B,U}}\right)}^{*}H_{u_{B,U}}^{b_{n}}F_{RF}^{b_{n}} \end{array}\right]$$

The effective network channel of $b_{n}$ includes not only the effective channels between the *BS* $b_{n}$ and *MSs* in the serving cell, but also the effective channels between the *BS* $b_{n}$ and *MSs* in the neighboring cells. Here, the total number of *MSs*, denoted by $u_{1,1},\;u_{1,2},\;\cdots ,\;u_{1,U},\;u_{2,1},\;\cdots ,\;u_{B,U}$, is BU because the number of neighboring *BSs* is B. A baseband precoder can be designed if we can obtain an inverse matrix of the effective network channel as follows:

(7) $$F_{BB}^{b_{n}}={\left({\tilde{H}}^{b_{n}}\right)}^{†}$$

where ${\left(\;\right)}^{†}$ denotes a pseudo inverse matrix operation. However, we cannot find the pseudo inverse matrix for ${\tilde{H}}^{b_{n}}\in {\mathbb{C}}^{BU\times U}$ because the effective network channel is given by a rectangular matrix with a large number of rows (${\tilde{H}}^{b_{n}}{\left({\tilde{H}}^{b_{n}}\right)}^{†}\neq I$). Thus, the zero-forcing (ZF) precoder, which is widely used in multiuser communication, cannot be obtained in a multicell multiuser environment. Note that the ZF precoder in a single-cell multiuser environment is given by an inverse (square) matrix of ${\tilde{H}}^{b_{n}}\in {\mathbb{C}}^{U\times U}$. In the Type-1 precoder, the ZF precoding technique for a single-cell multiuser is applied to each cell. It is expected that the Type-1 precoder cannot achieve a high capacity because it cannot reduce the IBI from neighboring cells.

When the Type-1 precoder is used, the achievable rate of the *MS* $u_{n,m}$ served by the *BS* $b_{n}$ is given by

(8) $$R_{u_{n,m}}^{b_{n}}=\log_{2}\left(1+\frac{\frac{\beta }{U}{\left|{\left(w_{RF}^{u_{n,m}}\right)}^{*}H_{u_{n,m}}^{b_{n}}F_{RF}^{b_{n}}f_{BB,u_{n,m}}^{b_{n}}\right|}^{2}}{\frac{\beta }{U}\sum_{g\neq m}{\left|{\left(w_{RF}^{u_{n,m}}\right)}^{*}H_{u_{n,m}}^{b_{n}}F_{RF}^{b_{n}}f_{BB,u_{n,g}}^{b_{n}}\right|}^{2}+\sum_{l\neq n}\left\{\frac{\beta }{U}\sum_{g=1}^{U}{\left|{\left(w_{RF}^{u_{n,m}}\right)}^{*}H_{u_{n,m}}^{b_{l}}F_{RF}^{b_{l}}f_{BB,u_{l,g}}^{b_{l}}\right|}^{2}\right\}+\sigma^{2}}\right)$$

where β denotes the total transmission power of the *BS*. Note that $b_{l}$ and $u_{l,g}$ denote the l-th *BS* and the g-th *MSs*, respectively, which are served by the $b_{l}-\mathrm{th}$ *BS*. In addition, $f_{BB,u_{n,m}}^{b_{n}}$ and $\sigma^{2}$ denote the (*U* * (*n* − 1) + *m*)-th column vector of $F_{BB}^{b_{n}}$ and the variance of Gaussian noise, respectively. The first term in the denominator represents the interference caused by adjacent beams in the serving cell, and the second term represents the interference caused by beams from neighboring *BSs*. The sum-rate and average-rate of the system are given by

(9) $$R_{sum}=\sum_{n=1}^{B}\sum_{m=1}^{U}R_{u_{n,m}}^{b_{n}},\;R_{avg}=\frac{R_{sum}}{BU}$$

### 2.2. Type-2 Precoder

In the Type-1 precoder, we cannot find a pseudo inverse matrix for the effective network channel. To solve the problem, we define an effective combined network channel as a square matrix combining all effective network channels ${\tilde{H}}^{b_{n}}$ in neighboring cells. The information on the effective network channel is shared by a backhaul link, as shown in Figure 2b. The effective combined network channel is defined by

(10) $${\tilde{H}}^{comb}=\left[{\tilde{H}}^{b_{1}},\;{\tilde{H}}^{b_{2}},\cdots ,{\tilde{H}}^{b_{B}}\right]=\left[\begin{array}{cccc} \begin{array}{c} {\left(w_{RF}^{u_{1,1}}\right)}^{*}H_{u_{1,1}}^{b_{1}}F_{RF}^{b_{1}}\\ {\left(w_{RF}^{u_{1,2}}\right)}^{*}H_{u_{1,2}}^{b_{1}}F_{RF}^{b_{1}}\\ \vdots \\ {\left(w_{RF}^{u_{1,U}}\right)}^{*}H_{u_{1,U}}^{b_{1}}F_{RF}^{b_{1}}\\ {\left(w_{RF}^{u_{2,1}}\right)}^{*}H_{u_{2,1}}^{b_{1}}F_{RF}^{b_{1}}\\ \vdots \\ {\left(w_{RF}^{u_{B,U}}\right)}^{*}H_{u_{B,U}}^{b_{1}}F_{RF}^{b_{1}} \end{array} & \begin{array}{c} {\left(w_{RF}^{u_{1,1}}\right)}^{*}H_{u_{1,1}}^{b_{2}}F_{RF}^{b_{2}}\\ {\left(w_{RF}^{u_{1,2}}\right)}^{*}H_{u_{1,2}}^{b_{2}}F_{RF}^{b_{2}}\\ \vdots \\ {\left(w_{RF}^{u_{1,U}}\right)}^{*}H_{u_{1,U}}^{b_{2}}F_{RF}^{b_{2}}\\ {\left(w_{RF}^{u_{2,1}}\right)}^{*}H_{u_{2,1}}^{b_{2}}F_{RF}^{b_{2}}\\ \vdots \\ {\left(w_{RF}^{u_{B,U}}\right)}^{*}H_{u_{B,U}}^{b_{2}}F_{RF}^{b_{2}} \end{array} & \cdots & \begin{array}{c} {\left(w_{RF}^{u_{1,1}}\right)}^{*}H_{u_{1,1}}^{b_{B}}F_{RF}^{b_{B}}\\ {\left(w_{RF}^{u_{1,2}}\right)}^{*}H_{u_{1,2}}^{b_{B}}F_{RF}^{b_{B}}\\ \vdots \\ {\left(w_{RF}^{u_{1,U}}\right)}^{*}H_{u_{1,U}}^{b_{B}}F_{RF}^{b_{B}}\\ {\left(w_{RF}^{u_{2,1}}\right)}^{*}H_{u_{2,1}}^{b_{B}}F_{RF}^{b_{B}}\\ \vdots \\ {\left(w_{RF}^{u_{B,U}}\right)}^{*}H_{u_{B,U}}^{b_{B}}F_{RF}^{b_{B}} \end{array} \end{array}\right]$$

Unlike (6), ${\tilde{H}}^{comb}\in {\mathbb{C}}^{BU\times BU}$ becomes a square matrix. In a Type-2 case, a ZF precoder for the effective combined network channel is obtained as follows:

(11) $${\left({\tilde{H}}^{comb}\right)}^{-1}=\left[\begin{array}{c} F_{BB}^{b_{1}}\\ F_{BB}^{b_{2}}\\ \vdots \\ F_{BB}^{b_{B}} \end{array}\right],\;F_{BB}^{b_{n}}\in {\mathbb{C}}^{U\times BU}$$

In a cellular system with the Type-2 precoder, the IBI reduction cannot be performed independently by each *BS* because the Type-2 precoder is operated on the network-level consisting of all neighboring *BSs*. In other words, during the data transmission period, all *BSs* should share data streams and transmit data streams simultaneously to all *MSs*. The number of beams that eventually formed at the *BS* is the same as that of all *MSs*, BU ($F_{RF}^{b_{n}}F_{BB}^{b_{n}}\in {\mathbb{C}}^{N_{BS}\times BU}$), which is much larger than U used in the first stage of the *RF* precoder design. The achievable rate of the *MS* $u_{n,m}$ served by the *BS* $b_{n}$ in the Type-2 precoder is given by

(12) $$R_{u_{n,m}}^{b_{n}}=\log_{2}\left(1+\frac{\sum_{l=1}^{B}\left\{\frac{\beta }{U}{\left|{\left(w_{RF}^{u_{n,m}}\right)}^{*}H_{u_{n,m}}^{b_{l}}F_{RF}^{b_{l}}f_{BB,u_{n,m}}^{b_{l}}\right|}^{2}\right\}}{\sum_{l=1}^{B}\left\{\frac{\beta }{U}\sum_{j\neq n\wedge g\neq m}{\left|{\left(w_{RF}^{u_{n,m}}\right)}^{*}H_{u_{n,m}}^{b_{l}}F_{RF}^{b_{l}}f_{BB,u_{j,g}}^{b_{l}}\right|}^{2}\right\}+\sigma^{2}}\right)$$

If interferences received from all neighboring *BSs* are cancelled with a perfect estimation of all effective network channels, the interference term in the denominator of (12) will become zero.

### 2.3. Type-3 Precoder

The Type-2 precoder requires a large network overhead because the precoder is operated on the network-level consisting of all neighboring *BSs*. To reduce the network overhead, the *BS* with a Type-3 precoder forms analog beams in the direction of not only the *MSs* in the serving cell but also the *MSs* in the neighboring cell, as shown in Figure 2c. In this case, the Type-3 precoder allows us to design the digital precoder of each *BS* independently. The *RF* precoder in the Type-3 precoder is given by

(13) $$F_{RF}^{b_{n}}=A_{BS}^{b_{n}}=[a_{BS}^{u_{1,1}}\left(\phi_{BS}^{u_{1,1}},\theta_{BS}^{u_{1,1}}\right),\;\cdots ,\;a_{BS}^{u_{1,U}}\left(\phi_{BS}^{u_{1,U}},\theta_{BS}^{u_{1,U}}\right),\;a_{BS}^{u_{2,1}}\left(\phi_{BS}^{u_{2,1}},\theta_{BS}^{u_{2,1}}\right),\;\cdots ,\;a_{BS}^{u_{U,U}}\left(\phi_{BS}^{u_{B,U}},\theta_{BS}^{u_{B,U}}\right)]$$

The effective network channel for each *BS* with the Type-3 precoder is given by (6). However, unlike the Type-1 precoder, the effective network channel for the *BS* $b_{n}$ with the Type-3 precoder, ${\tilde{H}}^{b_{n}}\in {\mathbb{C}}^{BU\times BU}$, becomes a square matrix because of the increase in the number of *RF* precoder. In the Type-3 case, a ZF precoder for the effective network channel is obtained as follows:

(14) $$F_{BB}^{b_{n}}={\left({\tilde{H}}^{b_{n}}\right)}^{-1}$$

Unlike the Type-2 case, the *BS* with the Type-3 precoder can operate independently during the data transmission period. In addition, the number of beams that eventually formed at the *BS* is the same as the number of *MSs* in the serving cell, U. Using (6) and (13), the effective network channel in the Type-3 case can be expressed as

(15) $$\begin{array}{l} {\tilde{H}}^{b_{n}}=D^{b_{n}}A_{BS}^{b_{n}}{\left(A_{BS}^{b_{n}}\right)}^{*}\\ {\left[D^{b_{n}}\right]}_{u_{l,g},u_{l,g}}=\sqrt{N_{BS}N_{MS}}\alpha_{u_{l,g}}^{}{\left(w_{RF}^{u_{l,g}}\right)}^{*}a_{MS}^{u_{l,g}}\left(\phi_{MS}^{u_{l,g}},\theta_{MS}^{u_{l,g}}\right) \end{array}$$

If l is equal to n and the *RF* combiner of the *MS* $u_{n,g}$ is correctly steered to the direction of the *BS* $b_{n}$, the equation ${\left(w_{RF}^{u_{n,g}}\right)}^{*}a_{MS}^{u_{n,g}}\left(\phi_{MS}^{u_{n,g}},\theta_{MS}^{u_{n,g}}\right)=1$ is always satisfied. In addition, the hybrid precoder satisfies the following power constraint:

(16) $${\left\|F_{RF}^{b_{n}}f_{BB,u_{n,m}}^{b_{n}}\right\|}^{2}=1$$

Then, the precoder in (14) can be rewritten as follows:

(17) $$\begin{array}{l} F_{BB}^{b_{n}}={\left({\tilde{H}}^{b_{n}}\right)}^{-1}\Lambda ={\left(D^{b_{n}}A_{BS}^{b_{n}}{\left(A_{BS}^{b_{n}}\right)}^{*}\right)}^{-1}\Lambda \\ f_{BB,u_{n,m}}^{b_{n}}={\left[F_{BB}^{b_{n}}\right]}_{:,u_{n,m}}={\left(D^{b_{n}}A_{BS}^{b_{n}}{\left(A_{BS}^{b_{n}}\right)}^{*}\right)}^{-1}{\left[\Lambda \right]}_{:,u_{n,m}} \end{array}$$

where Λ represents the diagonal matrix inserted to satisfy the power constraint in (16). ${\left[\Lambda \right]}_{:,u_{n,m}}$ denotes the (U * (*n −* 1) *+ m*)-th row vector of Λ. Using (13) and (17), (16) can be rewritten as

(18) $$\begin{array}{l} {\left\|F_{RF}^{b_{n}}f_{BB,u_{n,m}}^{b_{n}}\right\|}^{2}={\left(F_{RF}^{b_{n}}f_{BB,u_{n,m}}^{b_{n}}\right)}^{*}\left(F_{RF}^{b_{n}}f_{BB,u_{n,m}}^{b_{n}}\right)\\ ={\left[\Lambda \right]}_{:u_{n,m},:}{\left({\left(D^{b_{n}}\right)}^{*}\right)}^{-1}{\left({\left(A_{BS}^{b_{n}}\right)}^{*}A_{BS}^{b_{n}}\right)}^{-1}{\left(A_{BS}^{b_{n}}\right)}^{*}A_{BS}^{b_{n}}{\left({\left(A_{BS}^{b_{n}}\right)}^{*}A_{BS}^{b_{n}}\right)}^{-1}{\left(D^{b_{n}}\right)}^{-1}{\left[\Lambda \right]}_{::,u_{n,m}}\\ ={\left[\Lambda \right]}_{u_{n,m},u_{n,m}}^{2}{\left[D\right]}_{u_{m},u_{m}}^{-2}{\left({\left(A_{BS}^{b_{n}}\right)}^{*}A_{BS}^{b_{n}}\right)}_{u_{n,m},u_{n,m}}^{-1}=1 \end{array}$$

Using (15) and (18), ${\left[\Lambda \right]}_{u_{n,m},u_{n,m}}^{}$ can be expressed as

(19) $${\left[\Lambda \right]}_{u_{n,m},u_{n,m}}^{}=\sqrt{\frac{N_{BS}N_{MS}}{{\left({\left(A_{BS}^{b_{n}}\right)}^{*}A_{BS}^{b_{n}}\right)}_{u_{n,m},u_{n,m}}^{-1}}}\left|\alpha_{u_{n,m}}\right|$$

when the interference signals are completely removed by the Type-3 precoder, the achievable rate in (8) is given by

(20) $$R_{u_{n,m}}^{b_{n}}=\log_{2}\left(1+\frac{\frac{\beta }{U}{\left|{\left[\Lambda \right]}_{u_{n,m},u_{n,m}}^{}\right|}^{2}}{\sigma^{2}}\right)=\log_{2}\left(1+\frac{\beta }{U\sigma^{2}}\frac{N_{BS}N_{MS}{\left|\alpha_{u_{n,m}}\right|}^{2}}{{\left({\left(A_{BS}^{b_{n}}\right)}^{*}A_{BS}^{b_{n}}\right)}_{u_{n,m},u_{n,m}}^{-1}}\right)$$

### 2.4. Type-4 Precoder

A Type-4 precoder is proposed for the case where a hybrid beamformer is available at the *MS* as well as the *BS*. In the Type-4 case, the precoder at the *BS* removes only the interference from adjacent beams in the serving cell, while the combiner at the *MS* is designed to remove interference from neighboring cells. As shown in Figure 2d, the *BS* forms beams in the directions of *MSs* in the serving cell, while the *MSs* form beams in the direction of not only the serving *BS*, but also neighboring *BSs* that generate interference to *MSs* in the serving cell. For example, in Figure 2d, the *MS* $u_{1,1}$ forms beams in the directions of *BSs* $b_{1}$ and $b_{2}$. The precoder at the *BS* $b_{1}$ removes the inter-beam interference in the serving cell, while the combiner at the *MS* $u_{1,1}$ removes interference from neighboring *BS* $b_{2}$. In the Type-4 case, the *RF* precoder at the *BS* is given by

(21) $$F_{RF}^{b_{n}}=A_{BS}^{b_{n}}=[a_{BS}^{u_{n,1}}\left(\phi_{BS}^{u_{n,1}},\theta_{BS}^{u_{n,1}}\right),\;a_{BS}^{u_{n,2}}\left(\phi_{BS}^{u_{n,2}},\theta_{BS}^{u_{n,2}}\right),\;\cdots ,\;a_{BS}^{u_{n,U}}\left(\phi_{BS}^{u_{n,U}},\theta_{BS}^{u_{n,U}}\right)]$$

A digital precoding technique at the *BS* in the Type-4 case is the same as that in the Type-1 case. The *RF* combiner at the *MS* is given by

(22) $$W_{RF}^{u_{n,m}}=[a_{MS}^{b_{1},u_{n,m}}\left(\phi_{MS}^{b_{1},u_{n,m}},\theta_{MS}^{b_{1},u_{n,m}}\right),\;a_{MS}^{b_{2},u_{n,m}}\left(\phi_{MS}^{b_{2},u_{n,m}},\theta_{MS}^{b_{2},u_{n,m}}\right),\;\cdots ,\;a_{MS}^{b_{B},u_{n,m}}\left(\phi_{MS}^{b_{B},u_{n,m}},\theta_{MS}^{b_{B},u_{n,m}}\right)]$$

where $a_{MS}^{b_{l},u_{n,m}}\left(\phi_{MS}^{b_{l},u_{n,m}},\theta_{MS}^{b_{l},u_{n,m}}\right)\prime$ denotes the antenna-array response vector at the *MS* $u_{n,m}$ formed toward the *BS* $b_{l}$. The effective network channel of the *BS* $b_{n}$ in the Type-4 case is given by

(23) $${\tilde{H}}^{b_{n}}=\left[\begin{array}{c} {\left(w_{RF}^{u_{n,1}}\right)}^{*}H_{u_{1,1}}^{b_{n}}F_{RF}^{b_{n}}\\ {\left(w_{RF}^{u_{n,2}}\right)}^{*}H_{u_{1,2}}^{b_{n}}F_{RF}^{b_{n}}\\ \vdots \\ {\left(w_{RF}^{u_{n,U}}\right)}^{*}H_{u_{B,U}}^{b_{n}}F_{RF}^{b_{n}} \end{array}\right]$$

Unlike (6) in the Type-1 case, only *MS*s in the serving cell are considered in the effective network channel in (23). In the Type-4 case, the *MS* also forms an effective network channel as follows:

(24) $${\tilde{H}}_{u_{n,m}}=\left[\begin{array}{ccc} {\left(W_{RF}^{u_{n,m}}\right)}^{*}\sum_{g=1}^{U}\left(H_{u_{n,m}}^{b_{1}}f_{RF,u_{1,g}}^{b_{1}}\right) & \cdots & {\left(W_{RF}^{u_{n,m}}\right)}^{*}\sum_{g=1}^{U}\left(H_{u_{n,m}}^{b_{B}}f_{RF,u_{B,g}}^{b_{B}}\right) \end{array}\right]$$

where ${\tilde{H}}_{u_{n,m}}$ denotes an effective network channel of the *MS* $u_{n,m}$. The baseband combiner at the *MS* is designed as

(25) $$W_{BB}^{u_{n,m}}={\left({\tilde{H}}_{u_{n,m}}\right)}^{-1}$$

The number of analog beams required at the *BS* in the Type-4 case is U, which is much smaller than that in the Type-3 case. Thus, a smaller number of *RF* chains is required at the *BS* in the Type-4 case. However, the number of *RF* chains required at the *MS* in the Type-4 case increases from 1 to B. In the Type-4 case, the achievable rate of the *MS* $u_{n,m}$ in the serving the *BS* $b_{n}$ is given by

(26) $$R_{u_{n,m}}^{b_{n}}=\log_{2}\left(1+\frac{\frac{\beta }{U}{\left|W_{BB}^{u_{n,m}}{\left(W_{RF}^{u_{n,m}}\right)}^{*}H_{u_{n,m}}^{b_{n}}F_{RF}^{b_{n}}f_{BB,u_{n,m}}^{b_{n}}\right|}^{2}}{\frac{\beta }{U}\sum_{g\neq m}{\left|W_{BB}^{u_{n,m}}{\left(W_{RF}^{u_{n,m}}\right)}^{*}H_{u_{n,m}}^{b_{n}}F_{RF}^{b_{n}}f_{BB,u_{n,g}}^{b_{n}}\right|}^{2}+\sum_{l\neq n}\left\{\frac{\beta }{U}\sum_{g=1}^{U}{\left|W_{BB}^{u_{n,m}}{\left(W_{RF}^{u_{n,m}}\right)}^{*}H_{u_{n,m}}^{b_{l}}F_{RF}^{b_{l}}f_{BB,u_{l,g}}^{b_{l}}\right|}^{2}\right\}+\sigma^{2}}\right)$$

Unlike the Type-3 case, the baseband combiner of the *MS* is reflected in the achievable rate in the Type-4 case. As in (15), the effective network channel of the *BS* $b_{n}$ can be written as

(27) $$\begin{array}{l} {\tilde{H}}^{b_{n}}=D^{b_{n}}A_{BS}^{b_{n}}{\left(A_{BS}^{b_{n}}\right)}^{*}\\ A_{BS}^{b_{n}}=\left[a_{BS}^{u_{n,1}}\left(\phi_{BS}^{u_{n,1}},\theta_{BS}^{u_{n,1}}\right),\;a_{BS}^{u_{n,2}}\left(\phi_{BS}^{u_{n,2}},\theta_{BS}^{u_{n,2}}\right),\cdots ,\;a_{BS}^{u_{n,U}}\left(\phi_{BS}^{u_{n,U}},\theta_{BS}^{u_{n,U}}\right)\right] \end{array}$$

Note that the effective network channel in (27) considers only *MSs* in the serving cell. Using the result in (19), the diagonal matrix for the power constraint in the *BS* precoder can be expressed as

(28) $${\left[\Lambda_{BS}^{b_{n}}\right]}_{u_{n,m},u_{n,,m}}^{}=\sqrt{\frac{N_{BS}N_{MS}}{{\left({\left(A_{BS}^{b_{n}}\right)}^{*}A_{BS}^{b_{n}}\right)}_{u_{n,m},u_{n,m}}^{-1}}}\left|\alpha_{u_{n,m}}\right|$$

From (24), the effective network channel of the *MS* can be rewritten as

(29) $$\begin{array}{l} {\tilde{H}}_{u_{n,m}}={\left(A_{MS}^{u_{n,m}}\right)}^{*}A_{MS}^{u_{n,m}}D_{u_{n,m}}\\ A_{MS}^{u_{n,m}}=[a_{MS}^{b_{1,}u_{n,m}}\left(\phi_{BS}^{b_{1,}u_{n,m}},\theta_{BS}^{b_{1,}u_{n,m}}\right)a_{MS}^{b_{2},u_{n,m}}\left(\phi_{BS}^{b_{2},u_{n,m}},\theta_{BS}^{b_{2},u_{n,m}}\right)\;\cdots \;a_{MS}^{b_{B},u_{n,m}}\left(\phi_{BS}^{b_{B},u_{n,m}},\theta_{BS}^{b_{B},u_{n,m}}\right)]\\ {\left[D_{u_{n,m}}\right]}_{b_{n},b_{n}}=\sqrt{N_{BS}N_{MS}}\alpha_{b_{n},u_{n,m}}^{}\sum_{g=1}^{U}\left\{{\left(a_{BS}^{b_{n},u_{n,m}}\left(\phi_{BS}^{b_{n},u_{n,m}},\theta_{BS}^{b_{n},u_{n,m}}\right)\right)}^{*}f_{RF,u_{n,g}}^{b_{n}}\right\} \end{array}$$

The diagonal matrix for the power constraint in the baseband combiner of the *MS* can be obtained in a similar way, as follows:

(30) $${\left[\Lambda_{MS}^{u_{n,m}}\right]}_{b_{n},b_{n}}^{}=\sqrt{\frac{N_{BS}N_{MS}}{{\left({\left(A_{MS}^{u_{n,m}}\right)}^{*}A_{MS}^{u_{n,m}}\right)}_{b_{n},b_{n}}^{-1}}}\left|\alpha_{b_{n},u_{n,m}}^{}\sum_{g=1}^{U}\left\{{\left(a_{BS}^{b_{n},u_{n,m}}\left(\phi_{BS}^{b_{n},u_{n,m}},\theta_{BS}^{b_{n},u_{n,m}}\right)\right)}^{*}f_{RF,u_{n,g}}^{b_{n}}\right\}\right|$$

The effective network channels in (27) and (29) can be estimated independently at the *BS* and *MS*, respectively. Thus, the digital precoder at the *BS* and combiner at the *MS* can be designed independently in the Type-4 case. To obtain an achievable rate in the Type-4 precoder, the numerator in (26) is rewritten as

(31) $$\begin{array}{l} W_{BB}^{u_{n,m}}{\left(W_{RF}^{u_{n,m}}\right)}^{*}H_{u_{n,m}}^{b_{n}}F_{RF}^{b_{n}}f_{BB,u_{n,m}}^{b_{n}}\\ =W_{BB}^{u_{n,m}}{\left(W_{RF}^{u_{n,m}}\right)}^{*}a_{MS}^{b_{n,}u_{n,m}}\left(\phi_{BS}^{b_{n,}u_{n,m}},\theta_{BS}^{b_{n,}u_{n,m}}\right){\left[\Lambda_{BS}^{b_{n}}\right]}_{u_{n,m},u_{n,m}}\\ =\frac{1}{\sqrt{N_{BS}N_{MS}}\alpha_{b_{n},u_{n,m}}^{}\sum_{g=1}^{U}\left\{{\left(a_{BS}^{b_{n},u_{n,m}}\left(\phi_{BS}^{b_{n},u_{n,m}},\theta_{BS}^{b_{n},u_{n,m}}\right)\right)}^{*}f_{RF,u_{n,g}}^{b_{n}}\right\}}{\left[\Lambda_{MS}^{u_{n,m}}\right]}_{b_{n},b_{n}}{\left[\Lambda_{BS}^{b_{n}}\right]}_{u_{n,m},u_{n,,m}}^{}\\ =\sqrt{\frac{N_{BS}N_{MS}}{{\left({\left(A_{BS}^{b_{n}}\right)}^{*}A_{BS}^{b_{n}}\right)}_{u_{n,m},u_{n,m}}^{-1}{\left({\left(A_{MS}^{u_{n,m}}\right)}^{*}A_{MS}^{u_{n,m}}\right)}_{b_{n},b_{n}}^{-1}}}\left|\alpha_{b_{n},u_{n,m}}^{}\right| \end{array}$$

Then, the averaged achievable rate is given by

(32) $$R_{u_{n,m}}^{b_{n}}=\log_{2}\left(1+\frac{P}{U_{1}\sigma^{2}}\frac{N_{BS}N_{MS}{\left|\alpha_{b_{n},u_{n,m}}^{}\right|}^{2}}{{\left({\left(A_{BS}^{b_{n}}\right)}^{*}A_{BS}^{b_{n}}\right)}_{u_{n,m},u_{n,m}}^{-1}{\left({\left(A_{MS}^{u_{n,m}}\right)}^{*}A_{MS}^{u_{n,m}}\right)}_{b_{n},b_{n}}^{-1}}\right)$$

From (32), one can see that the averaged achievable rate in the Type-4 precoder is affected by not only the array response matrix of the *BS*, but also the array-response matrix of the *MS*. 

In summary, in this section, we propose four different precoding techniques to reduce the IBI in mm-wave cellular systems with a hybrid beamformer. The Type-1 precoder is simple because the precoding technique developed for a single-cell multiuser environment is applied to each cell. However, in this case, we cannot reduce the interference from neighboring cells. The Type-2 precoder can reduce interference from both the serving cell and neighboring cells because the precoder is designed for the combined network consisting of all neighboring cells. However, in this case, all *BSs* should share data streams, increasing the network overhead. In the Type-3 precoder, the *RF* precoder is extended such that the digital precoder can be designed independently by each *BS*. However, in this case, the number of *RF* chains that are required at the *BS* increases as the number of neighboring cells increases. The Type-4 precoder can be used when a hybrid beamformer is available at the *MS* as well as at the *BS*. In this case, the number of *RF* chains required at the *BS* is reduced to a single-cell case; however, the number of *RF* chains required at the *MS* increases by the number of neighboring cells.

### 2.5. Channel Estimation

So far, we assumed that the channel information required for the precoder design is known perfectly. However, the channels between the *MS* and neighboring *BSs*, as well as the channel between the *MS* and the serving *BS*, should be estimated beforehand for the design of the proposed precoder. The processing time required for channel estimation in the proposed approach increases proportionally to the product of the number of neighboring cells and the number of Tx beams. In order to reduce the processing time significantly, we apply the cell and beam reference signal (CBRS) technique to the effective network channel estimation for the design of the proposed precoder [18]. Here, we assume that the *RF* precoder in the *BS* and the *RF* combiner in the *MS* are successfully obtained after completion of the first stage in the analog domain. 

In the CBRS technique, multiple beams are transmitted simultaneously from neighboring *BSs* (including the serving cell) to reduce the processing time for channel estimation. In order to estimate all channels between the *MS* and neighboring *BSs* with multiple beams, CBRSs are simultaneously transmitted from multiple beams of neighboring *BSs*. The CBRS carries a cell ID (CID) as well as a beam ID (BID). If the CBRS carries only BID information, it will be impossible to determine which cell had transmitted the BID. Therefore, a large number of different sequences (CID × BID) need to be generated. In the proposed CBRS technique, the BID is designed in conjunction with CID because the BID must be detected in a multicell environment. The hierarchical structure of CBRS gives the advantage of enabling the reuse of the same BIDs in neighboring cells, enabling us to generate a large set of sequences. 

The CBRS technique can be viewed as a combination of a Chu sequence and a polyphase sequence. In the CBRS technique, a sequence is generated by mapping the CID to the root index of the Chu sequence, and the BID to the index of the polyphase sequence. The Chu sequence is selected as a base sequence because it is widely used in the design of synchronization signals and the random access preamble in Long Term Evolution (LTE) systems owing to its good correlation property [27]. A combination of a Chu sequence and a polyphase sequence is selected for CBRS because these signals can be transmitted simultaneously with minimal IBI in a multicell environment. The CBRS is defined by the product of a prime-length Chu sequence and a polyphase sequence in the frequency domain as follows:

(33) $$\begin{array}{l} S_{c,i}(k)=Z_{c}(k)P_{i}(k)\\ Z_{c}(k)=e^{\frac{j\pi ck(k+1)}{N}},\;P_{i}(k)=e^{\frac{-j2\pi iLk}{N}},\;k=0,1,\ldots ,N-1 \end{array}$$

where $Z_{c}(k)\;\mathrm{and}\;P_{i}(k)$ denote a prime-length Chu sequence and polyphase sequence, respectively. In addition, $c\in N_{C},\;i\in N_{I},\;N_{C}=\left\{1,\;2,\;\ldots ,\;N_{C}-1\right\},\;\mathrm{and}\;N_{I}=\left\{0,\;1,\;\ldots ,\;N_{I}-1\right\}$ denote the root index of the Chu sequence, index of polyphase sequence, set of CIDs, and set of BIDs, respectively. $N_{C}$, $N_{I}$, and N denote the number of available CIDs, BIDs, and sequence length, respectively. L is a parameter used to avoid the incorrect detection of the cell ID when there is a symbol timing offset (STO).

The beams are generated by the *RF* precoder at the *BS*, which is obtained after completion of the first stage. The signal received at the *MS* after CBRS transmission is given by

(34) $$y_{u_{n,m}}^{}={\left(w_{RF}^{u_{n,m}}\right)}^{*}\left\{\sum_{l=1}^{B}\left(H_{u_{n,m}}^{b_{l}}F_{RF}^{b_{l}}s^{b_{l}}\right)+n_{u_{n,m}}\right\}$$

Note that only the *RF* precoder is used at the *BS*. The baseband precoder will be designed in the second stage using the information of the estimated channels. Here, $s^{b_{l}}$ denotes the CBRSs that are transmitted from the *BS* $b_{l}$. The *MS* estimates the effective network channel using the correlation property of the CBRS. The highest peak occurs when the reference BID $\left(i^{\prime }\right)$ is matched with the transmitted BID (i). The correlator output becomes zero when the reference BID is not matched with the transmitted BID. The correlator output becomes a small value ($\frac{1}{\sqrt{N}}$) when the reference CID and BID $\left(\;c^{\prime },\;i^{\prime }\right)$ is not matched with the transmitted CID and BID (c,i). Using these properties, the *MS* can estimate all effective channels in neighboring cells by correlating the received signal with all possible reference CIDs and BIDs.

If the total number of beams to be generated in neighboring *BSs* is smaller than the number of BIDs $\left(N_{I}\right)$ generated by the CBRS, it will be advantageous to allocate the same CID to the neighboring *BSs*. In this case, no IBI will occur owing to the ideal correlation property, which allows us to estimate all channels accurately. However, if the total number of beams to be generated in neighboring *BSs* is larger than the number of BIDs $\left(N_{I}\right)$, it will be advantageous to allocate the same CID to the *BSs* located near the serving *BS*, and to allocate different CIDs to the *BSs* located far way. In this case, the interference term ($\frac{1}{\sqrt{N}}$), which is generated by the *BS* located far way, will be reduced further owing to the path loss.

## 3. Simulation

In this section, we evaluate the performance of the proposed hybrid beamforming technique for the reduction of IBI in mm-wave cellular systems. To do this, we perform computer simulations in a simple scenario shown in Figure 2. Here, we assume that four *MSs* are served by each *BS* in a three-cell environment. The *BS* and *MS* employ 8 × 8 and 4 × 4 UPAs, respectively. We also assumed that *MSs* are located randomly in a cell, and that a single LoS path is present between the *BS* and *MS*. The azimuth and elevation angles of AoD at the *BS* follow uniform distributions of (0, π) and (0, π/2), respectively. The path gain and the transmission power of *BS* are all normalized to 1. More sophisticated simulation can be performed with an accurate mm-wave channel model under realistic assumption [28].

**Figure 3:**
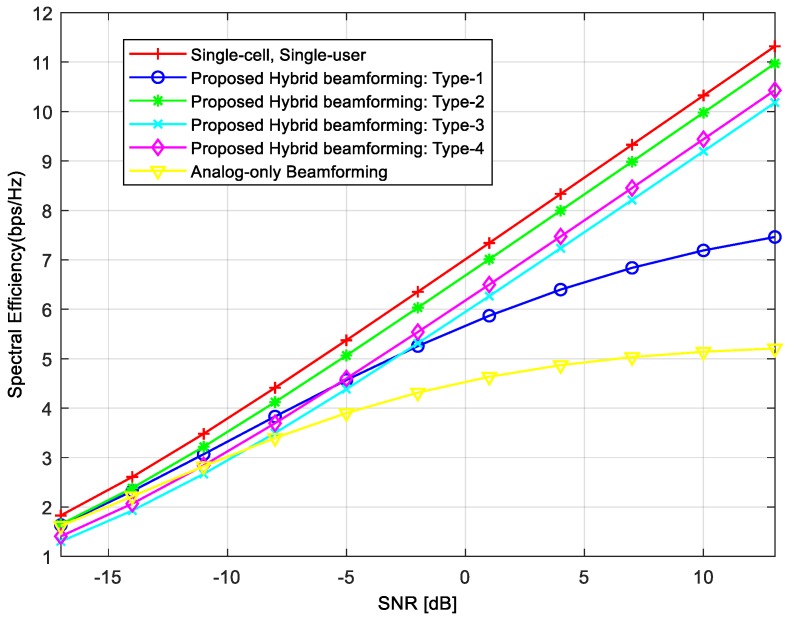
Achievable rates of proposed hybrid beamforming techniques.

In the first numerical simulation, we compared the averaged achievable rates per user for different hybrid beamforming techniques. In Figure 3, the legend “analog-only beamforming” refers to the case where only the *RF* precoders are used. In this case, beams are formed in the direction of *MSs* to maximize the desired signal power. However, its averaged achievable rate per user is the lowest because, in this case, we did not consider IBI. We can achieve the highest averaged achievable rate when we used the beamformer with “Single-cell, Single-user.” In this case, the best performance is achieved because there is no IBI from the serving cell or neighboring cells. The averaged achievable rates of the proposed hybrid beamforming techniques lie between “Analog-only Beamforming” and “single-cell, single-user”. The Type-1 precoder has the lowest achievable rates among the proposed techniques, and its achievable rate does not increase linearly as SNR increases because the interference from neighboring *BSs* is not removed. Note that the Type-1 precoder removes IBI in the serving cell, and does not consider IBI from neighboring cells. The achievable rates of Type-2, Type-3, and Type-4 precoders increase linearly as SNR increases, because the IBI from neighboring cells as well as the serving cell is reduced. The achievable rate is higher in the order of Type-2, Type-3, and Type-4 precoders (i.e., the highest is Type-2).

**Figure 4:**
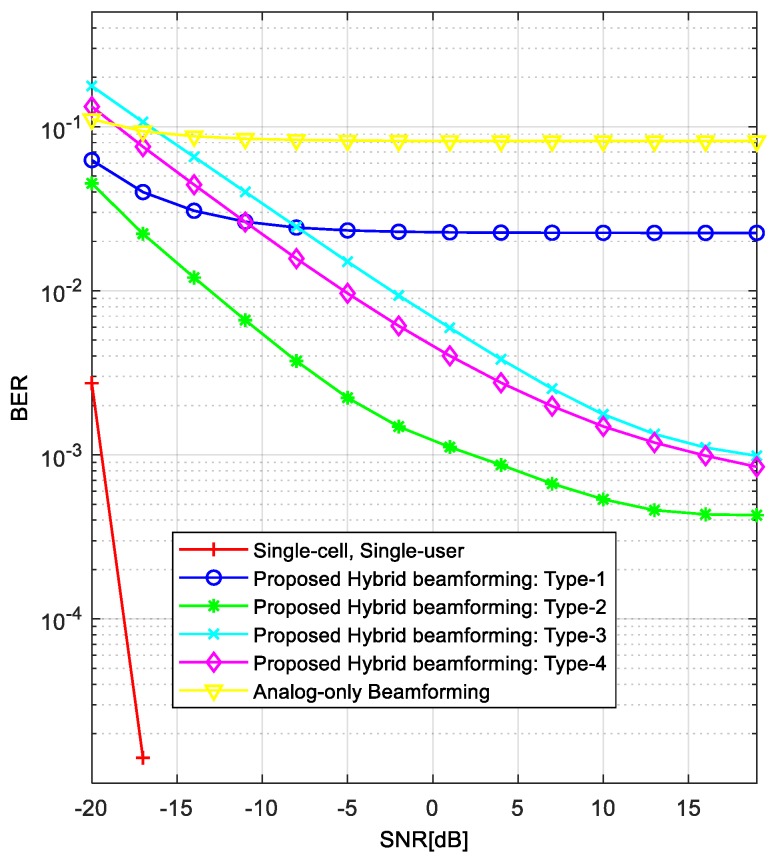
BER comparison of proposed hybrid beamforming techniques.

Figure 4 compares the BER performance of different hybrid beamforming techniques in the same environment as in Figure 3. By comparing these two figures, we can see that the BER performance in Figure 4 is consistent with the averaged achievable rates in Figure 3. This is because the achievable rate per user increases as the interference decreases, resulting in a decreased BER. The “analog-only beamforming” case shows the worst performance (error floor) because IBI is not considered. In addition, the “Single-cell, Single-user” case shows the best performance because there is no IBI. The Type-1 precoder shows the lowest performance (error floor) of the proposed techniques because it cannot reduce the IBI from neighboring cells. The BER performance is better in the order of Type-2, Type-3, and Type-4 precoders (i.e., the best is Type-2). The reason for which the BER curves show noticeable performance gaps compared with the achievable rates is that the BER curve reflects the worst case more significantly. For the BER simulation, we randomly selected the values of AoAs and AoDs between *BSs* and *MSs*. In the process of averaging the error rates, the lowest error rate usually dominates the averaged BER performance.

**Figure 5:**
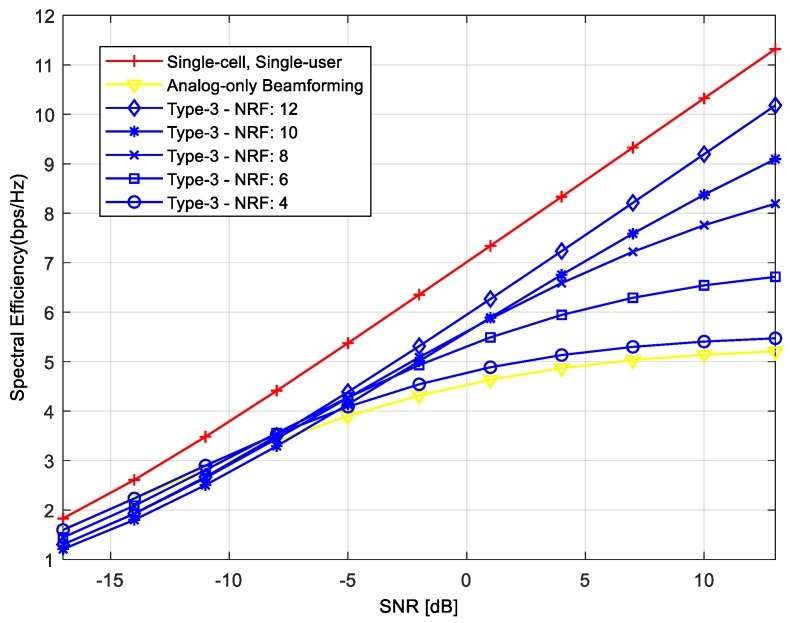
Achievable rates of Type-3 precoder when the number of radio frequency (*RF*) chains varies.

Type-1, Type-2, and Type-4 precoders require at least *U RF* chains to serve *U MSs* simultaneously in the serving cell. However, the number of *RF* chains in Type-3 precoder needs to be equal to or greater than the number of total *MSs* in neighboring cells to remove IBI. In the cellular system with 12 *MSs* in 3 neighboring cells, 12 *RF* chains are required for each *BS* to remove IBI. If the number of *RF* chains is smaller than 12, performance may degrade due to the remaining IBI. Figure 5 shows the achievable rate of Type-3 precoder when the number of *RF* chains varies. In simulation, the number of *RF* chains is changed from 4 to 12. As can be seen in the figure, the achievable rate decreases as the number of *RF* chains decreases. When the number of *RF* chains is 4, the performance of Type-3 precoder is similar to that of “Analog-only Beamforming” because Type-3 precoder cannot remove the IBI due to the insufficient number of *RF* chains.

**Figure 6:**
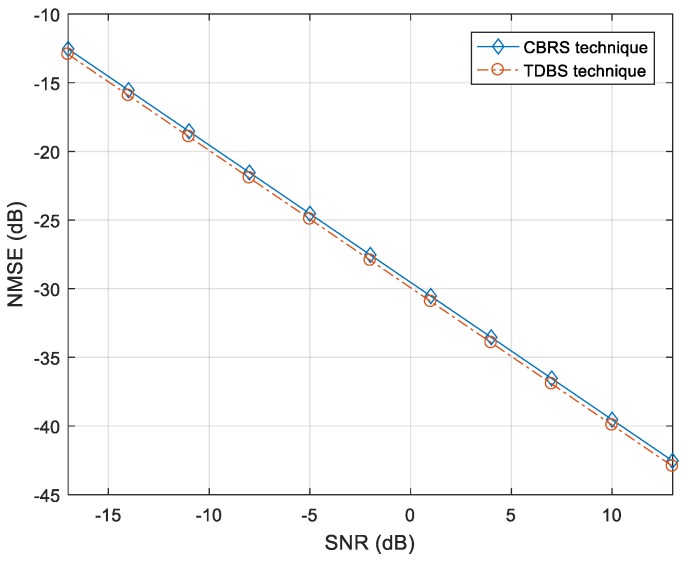
Performance comparison of channel estimation between cell and beam reference signal (CBRS) and time-division beam switching (TDBS) techniques.

Figure 6 shows the performance comparison when we used the CBRS technique and the time-division beam switching (TDBS) technique for channel estimation in the environment of Figure 2. In the CBRS technique, multiple beams with the same CID but different BIDs are transmitted simultaneously from neighboring *BSs*. In the TDBS technique, single Tx beams are individually transmitted from the *BS* until all of the Tx beams are transmitted. This process is repeated for neighboring *BSs*. The processing time required for beam transmission in TDBS increases proportionally to the product of the number of Tx beams and the number of neighboring *BSs*. As can be seen in Figure 6, the performances of two different channel estimation techniques are similar. It can be seen that the CBRS technique allows us to estimate the effective network channel with a minimal IBI in a multicell environment.

In Table 1, we compare the processing times required for channel estimation in the CBRS and TDBS techniques. Here, it is assumed that the subcarrier spacing Δf=75 kHZ and the symbol time $T_{S}=\frac{1}{75\;\text{kHZ}}≃13.33$ μs [13]. In the CBRS technique, the processing time required for channel estimation is one orthogonal frequency-division multiplexing (OFDM) symbol period because the CBRSs are transmitted simultaneously from neighboring *BSs* with multiple beams. Here, we ignore the time required to calculate the value of the channel estimate. The processing time required for beam transmission in the TDBS technique increases as the number of Tx beams and the number of neighboring *BSs* increase. When the TDBS technique is used for channel estimation and Type-1/Type-2/Type-4 precoders are used for interference reduction, the processing time required for channel estimation is increased by B×U because each *BS* needs to send one beam at a time to U *MSs* in the serving cell. In the Type-3 case, the processing time is increased by ${\left(B\right)}^{2}\times U$ because each *BS* needs to send beams in the direction of not only *MSs* in the serving cell but also *MSs* in neighboring cells. For example, when B=3 and U=5, the TDBS technique with the Type-1 and Type-3 precoders requires processing times that are 15 and 45 times longer, respectively, than with the CBRS technique.

Table 2 compares the sizes of the matrix and computational complexities required for proposed precoders. Since the digital precoders are designed using the estimated effective network channel matrices, the computational complexity of the proposed precoder is proportional to the size of the effective network channel matrices. As shown in Table 2, Type-2 and Type-3 precoders require the highest computational complexity. The followings are Type-1 and Type-4 precoders. The computational complexity is important in sensor networks because processing resources are constrained in most sensor nodes. The proposed precoders except Type-4 precoder do not require processing resources at the sensor node. All the computations required for the design of precoder are performed at the *BS*. The *MS* needs only to feedback the channel information to the *BS*. However, in Type-4 precoder, the *MS* needs processing resources to remove the IBI from adjacent cells. Note that, in the Type-4 case, the precoder at the *BS* removes only the interference from adjacent beams in the serving cell, while the combiner at the *MS* is designed to remove interference from adjacent cells. Table 2 also shows computational complexities required for the *BS* and *MS* (sensor node) when the proposed precoders are used. As can be seen in Table 2, all the computations required for the design of precoder (except Type-4 precoder) are performed at the *BS*. Thus, Type-1, Type-2, and Type-3 precoders can be easily applied to sensor networks because no additional hardware and processing resources are not required for sensor nodes. However, it may be difficult to apply the Type-4 precoder to typical sensor networks because the Type-4 precoder requires the hybrid beamformer and processing resources in sensor nodes.

**Figure 7:**
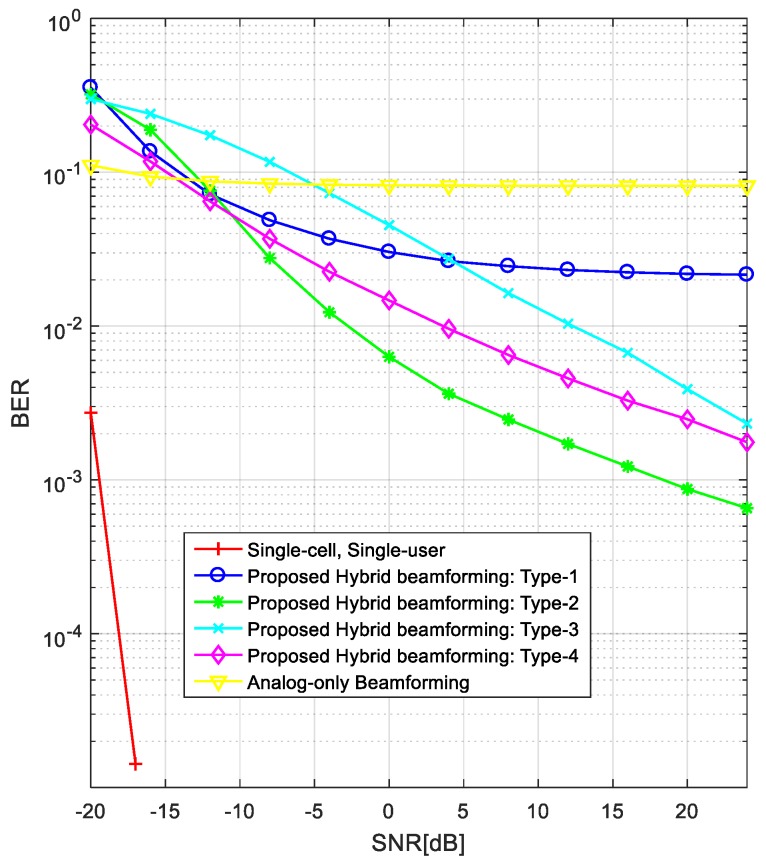
Comparison of BERs of proposed hybrid beamforming techniques when the estimated channel is used.

Figure 7 compares the BER performances of different hybrid beamforming techniques when we used the channel estimated by the CBRS technique. As can be seen in this figure, the BER performances of the proposed hybrid beamforming techniques are slightly degraded owing to the channel estimation error, compared with the results in Figure 4. The channel estimation error comes from the noise part in the received signal because mobility is not considered in this simulation. However, the overall trend in the performance is similar. Note that the BER performances of “Analog-only Beamforming” and “Single-cell, Single-user” are the same as in Figure 4 because the channel information is not used in these cases.

## 4. Conclusions

In this paper, we proposed digital precoding techniques for mm-wave systems with a hybrid beamformer, which can reduce the IBI received from neighboring cells as well as adjacent beams in the serving cell. We performed simulations, and the results show that while the Type-1 precoder is simple, it cannot reduce the IBI from neighboring cells, resulting in an error floor in BER performance. They also show that Type-2/Type-2/Type-3 precoders can achieve high achievable rates or low BERs because they can reduce the IBI from the serving cell and neighboring cells. The advantages and disadvantages of the precoders can be summarized as follows. In the Type-2 case, the number of *RF* chains required at the *BS* is the same as in a single-cell case. However, all neighboring *BSs* should share data streams, increasing the network overhead. In the Type-3 case, the digital precoder can be designed independently by each *BS*. However, the number of *RF* chains required at the *BS* increases. In the Type-4 case, the number of *RF* chains required at the *BS* is reduced to that of a single-cell case. However, the number of *RF* chains required at the *MS* increases. In addition, we showed that the processing times required for the estimation of the effective combined network channel can be significantly reduced using the CBRS technique. Finally, we showed that the proposed precoders (Type-2/Type-2/Type-3) with the estimated effective network channel can significantly reduce the IBI in mm-wave cellular systems. As a future work, the performance of the hybrid precoder needs to be analyzed in the environment where *MSs* move.

## Figures and Tables

**Table 1:** Processing time required for channel estimation in the proposed and conventional techniques.

		Processing Time	Example
CBRS technique		$T_{S}$	13.33 μs
TDBS technique	Type-1 precoder Type-2 precoder Type-4 precoder	$T_{S}\times B\times U$	200 μs
	Type-3 precoder	$T_{S}\times B^{2}\times U$	600 μs

**Table 2:** Computational complexities required for the design of the proposed precoders at base station (*BS*) and mobile station (*MS*).

		Type-1	Type-2	Type-3	Type-4
*BS*	Matrix dimension of ${\tilde{H}}^{b_{n}}$	BU×U	BU×BU	BU×BU	U×U
	Computational complexity for $F_{BB}^{b_{n}}$ calculation	$O({\left(BU\right)}^{2}U)$	$O({\left(BU\right)}^{3})$	$O({\left(BU\right)}^{3})$	$O(U^{3})$
*MS*	Matrix dimension of ${\tilde{H}}_{u_{n,m}}$	-	-	-	B×B
	Computational complexity for $W_{BB}^{u_{n,m}}$ calculation	-	-	-	$O(B^{3})$
